# Novel candidate genes for ECT response prediction—a pilot study analyzing the DNA methylome of depressed patients receiving electroconvulsive therapy

**DOI:** 10.1186/s13148-020-00891-9

**Published:** 2020-07-29

**Authors:** Nicole Moschny, Tristan Zindler, Kirsten Jahn, Marie Dorda, Colin F. Davenport, Lutz Wiehlmann, Hannah B. Maier, Franziska Eberle, Stefan Bleich, Alexandra Neyazi, Helge Frieling

**Affiliations:** 1 Laboratory for Molecular Neuroscience, Department of Psychiatry, Social Psychiatry and Psychotherapy, Hannover Medical School, Carl-Neuberg-Str. 1, Hannover, 30625 Germany; 2 Center for Systems Neuroscience, HGNI, University of Veterinary Medicine Hannover, Bünteweg 2, 30559 Hannover, Germany; 3 Department of Psychiatry, Social Psychiatry and Psychotherapy, Hannover Medical School, Carl-Neuberg-Str. 1, 30625 Hannover, Germany; 4 Research Core Unit Genomics, Hannover Medical School, Carl-Neuberg-Str. 1, 30625 Hannover, Germany

**Keywords:** Depression, DNA methylation, Electroconvulsive therapy, EWAS, Personalized medicine, Response prediction, Single-nucleotide polymorphism, RNF213, Ubiquitin, Autophagy

## Abstract

**Background:**

Major depressive disorder (MDD) represents a serious global health concern. The urge for efficient MDD treatment strategies is presently hindered by the incomplete knowledge of its underlying pathomechanism. Despite recent progress (highlighting both genetics and the environment, and thus DNA methylation, to be relevant for its development), 30–50% of MDD patients still fail to reach remission with standard treatment approaches. Electroconvulsive therapy (ECT) is one of the most powerful options for the treatment of pharmacoresistant depression; nevertheless, ECT remission rates barely reach 50% in large-scale naturalistic population-based studies. To optimize MDD treatment strategies and enable personalized medicine in the long- term, prospective indicators of ECT response are thus in great need. Because recent target-driven analyses revealed DNA methylation baseline differences between ECT responder groups, we analyzed the DNA methylome of depressed ECT patients using next-generation sequencing. In this pilot study, we did not only aim to find novel targets for ECT response prediction but also to get a deeper insight into its possible mechanism of action.

**Results:**

Longitudinal DNA methylation analysis of peripheral blood mononuclear cells isolated from a cohort of treatment-resistant MDD patients (*n* = 12; time points: before and after 1st and last ECT, respectively) using a TruSeq-Methyl Capture EPIC Kit for library preparation, led to the following results: (1) The global DNA methylation differed neither between the four measured time points nor between ECT responders (*n* = 8) and non-responders (*n* = 4). (2) Analyzing the DNA methylation variance for every probe (=1476812 single CpG sites) revealed eight novel candidate genes to be implicated in ECT response (protein-coding genes: *RNF175*, *RNF213*, *TBC1D14*, *TMC5*, *WSCD1*; genes encoding for putative long non-coding RNA transcripts: *AC018685.2*, *AC098617.1*, *CLCN3P1*). (3) In addition, DNA methylation of two CpG sites (located within *AQP10* and *TRERF1*) was found to change during the treatment course.

**Conclusions:**

We suggest ten novel candidate genes to be implicated in either ECT response or its possible mechanism. Because of the small sample size of our pilot study, our findings must be regarded as preliminary.

## Background

The World Health Organization [1] states major depressive disorder (MDD) to be one of the most prevalent mental diseases worldwide. Due to the high number of affected individuals (> 322 million), efficient treatment strategies are required. This need is being challenged by the insufficient knowledge of MDD’s underlying pathophysiology.

Research from recent decades reports nature and nurture to both be relevant for disease development: One’s individual genetic constitution provides a baseline for the vulnerability to certain diseases, but additional environmental factors are often mandatory to provoke their onset [2, 3]. This phenomenon is mediated by epigenetics, i.e., molecular mechanisms (such as DNA methylation (DNAm) and histone modifications) that modulate gene transcription without interfering with the DNA sequence itself [4–7]. In the case of MDD, animal experiments found the stress reactivity of rodent pups to be associated with their mother’s postnatal grooming behavior. In this context, hippocampal brain cells of neglected animals (in comparison with the pups being intensively cared for) showed a higher DNAm in gene regions encoding the glucocorticoid receptor [8, 9]. As a part of the hypothalamic-pituitary-adrenal (HPA) axis (our central stress response system) disturbances of the latter protein (together with other irregularities) have been suggested to be a cause for the lowered stress resilience found in depressed patients [10–12]. The importance of epigenetics for MDD is further underlined by Fuchikami et al., who distinguished depressed subjects from healthy controls simply by analyzing the DNAm of brain-derived neurotrophic factor (BDNF) [13]. BDNF is a neurotrophin shown to be implicated in various neuropsychiatric disorders, including MDD [14–16].

Despite this growing body of knowledge, treatment approaches for depression leave much to be desired: The proportion of MDD patients that fail to achieve full remission upon standard medication (30–50%) is still unsatisfyingly high [17]. Electroconvulsive therapy (ECT) has proven superior efficacy and is, therefore, considered to be one of the most powerful options for the treatment of pharmacoresistant depression [18, 19]. However, in naturalistic population-based community-setting studies, ECT remission rates barely reach 50% [20, 21]. To prevent medication failure at baseline and ensure patient-tailored treatment in the long-term, biomarkers predicting ECT response are thus of compelling need. Merely a few clinical characteristics (like age or psychotic symptoms, for instance [21–24]) serve as a guide for treatment-decision making, but due to MDD’s heterogeneity (compromising various subgroups and a broad spectrum of symptoms), a whole set of biomarkers will be required [25]. In this context, a few biological markers have been recently proposed, as the catechol-O-methyltransferase (*COMT*) Val158Met (rs4680) [26–28] or the dopamine receptor D2 (*DRD2*) C957T (rs6277) polymorphisms [29, 30]. In the field of epigenetics, our group recently found the DNAm of p11’s promoter (a protein implicated in BDNF production [31]) to reliably predict ECT response in two cohorts of MDD patients [32]. However, none of these targets is likely to reach sufficient sensitivity and specificity to act as an accurate predictor of ECT response alone and other studies on DNAm and ECT are missing [25, 33].

To find further indicators for ECT response prediction, we investigated the methylome of peripheral blood mononuclear cells (PBMCs) isolated from depressed patients undergoing a course of ECT. Most studies conduct their experiments in a target-driven manner and only investigate processes already known to be implicated in MDD. These hypothesis-based analyses provide an essential contribution to the field of biomarker research, though more data-driven approaches are still required so as to not overlook substantial ECT-related information. To address this issue, we used an Illumina EPIC Kit for our study, allowing an analysis of > 3.3 million CpGs located within regions known to be generally implicated in epigenetic mechanisms (such as CpG islands, promoter regions, and open chromatin). We thereby aimed (1) to identify novel targets for the prediction of ECT response and (2) to get a deeper insight into ECT’s general mechanisms.

## Results

### Patients’ clinical baseline characteristics

Patients’ clinical baseline characteristics are depicted in Table 1. After treatment completion, 10 patients (out of 17) responded to ECT. Four patients had minimally heightened levels of leukocytes (11.2–12.4 × 10^3^/μl), but no signs of infection (i.e., elevated C-reactive protein measures (CRP)). Patients were under medication while receiving ECT, but none were treated with immunomodulatory drugs. During ECT, patients were anesthetized with methohexital (mean = 128.2(± 53.3) mg, minimum = 90 mg, maximum = 250 mg) and remifentanil (89.7(± 49.8) mg, 30 mg, 200 mg) and received succinylcholine for muscle relaxation (114.1(± 45.0) mg, 60 mg, 200 mg). Responders (R) and non-responders (NR) differed only in their body mass index (BMI) (*t* test, *p* = 0.015, *T* = − 2.736, *R* = 28.4 ± 4.8, NR = 22.4 ± 4.8).

**Table 1:** Patients’ clinical baseline characteristics (*n* = 17)

		Whole cohort (*n* = 17)	Responders (*n* = 10)	Non-responders (*n* = 7)
Demographics				
Age in years, mean (±SD; range)		53.9 (± 16.7; 20–76)	57.1 (± 9.7; 43–70)	49.3 (± 23.7; 20–76)
Gender, *n* (%)	Female	10 (58.5%)	6 (60.0%)	4 (57.1%)
	Male	7 (41.2%)	4 (40.0%)	3 (42.9%)
Body mass index, mean (±SD; range)		25.9 (± 5.2; 17–39)	28.4 (± 4.8; 23–39)*	22.4 (± 4.5; 17–30)*
Smokers, *n* (%)	Yes	7 (43.8%)	6 (60.0%)	1 (14.3%)
Psychometric characteristics				
Age at diagnosis in years, mean (±SD; range)		33.6 (± 17.1; 14–74)	31.3 (± 14.0; 14–53)	36.8 (± 21.5; 18–74)
Current episode in weeks, mean (±SD; range)		36.3 (± 33.6; 3–124)	35.0 (± 38.7; 3–124)	39.0 (± 25.2; 16–68)
BDI, mean (±SD; range)		36.4 (± 10.9; 16–56)	35.3 (± 12.1; 16–56)	38.3 (± 9.2; 24–52)
MADRS, mean (±SD; range)		32.8 (± 10.3; 12–45)	33.8 (± 12.5; 12–45)	31.5 (± 7.4; 24–45)
MMSE, mean (±SD; range)		28.5 (± 2.6; 21–30)	28.0 (± 3.3; 21–30)	29.2 (± 1.3; 27–30)
Psychotic symptoms, *n* (%)	Yes	5 (29.4%)	3 (30.0%)	2 (28.6%)
Suicidality, *n* (%)	Yes	3 (17.6%)	0 (0.0%)	3 (42.9%)
Medication				
Antidepressant drugs, *n* (%)	Yes	17 (100.0%)	10 (100.0%)	7 (100.0%)
Benzodiazepines, *n* (%)	Yes	11 (64.7%)	7 (70.0%)	4 (57.1%)
Antipsychotic drugs, *n* (%)	Yes	11 (64.7%)	8 (80.0%)	3 (42.9%)
Lithium, *n* (%)	Yes	3 (17.6%)	1 (10.0%)	2 (28.6%)
Clinical parameters				
Leukocytes in × 10^3^/μl, mean (±SD; range)		7.6 (± 2.8; 3.5–12.4)	8.6 (± 2.4; 6.4–12.4)	6.2 (± 2.8; 3.5–12.1)

Clinical baseline characteristics of treatment-resistant depressed patients receiving a course of ECT (whole cohort vs. responders/non-responders), presented as mean (±standard deviation (SD); range (= minimum–maximum)) or quantity (absolute and percentual, *n* (%))

*BDI* beck depression inventory, *MADRS* Montgomery-*Å*sberg depression rating scale, *MMSE* mini-mental state examination

*p < 0.05

As described in the methods section, patients were excluded from the analysis if the DNAm values of at least one time point were missing (respectively). The clinical baseline characteristics of these patients (*n* = 12) are reported in Supplementary Table S1. In this subgroup, ECT responders and non-responders differed in their number of total leukocytes (*t* test, *p* = 0.048, *T* = **−** 2.249, *R* = 8.4 ± 2.2, NR = 5.7 ± 1.2) and their current episode duration (*t* test, *p* = 0.026, *T* = 2.948, *R* = 24.8 ± 15.2, NR = 60.0 ± 11.3).

### ECT and DNA methylation

**Fig. 1:**
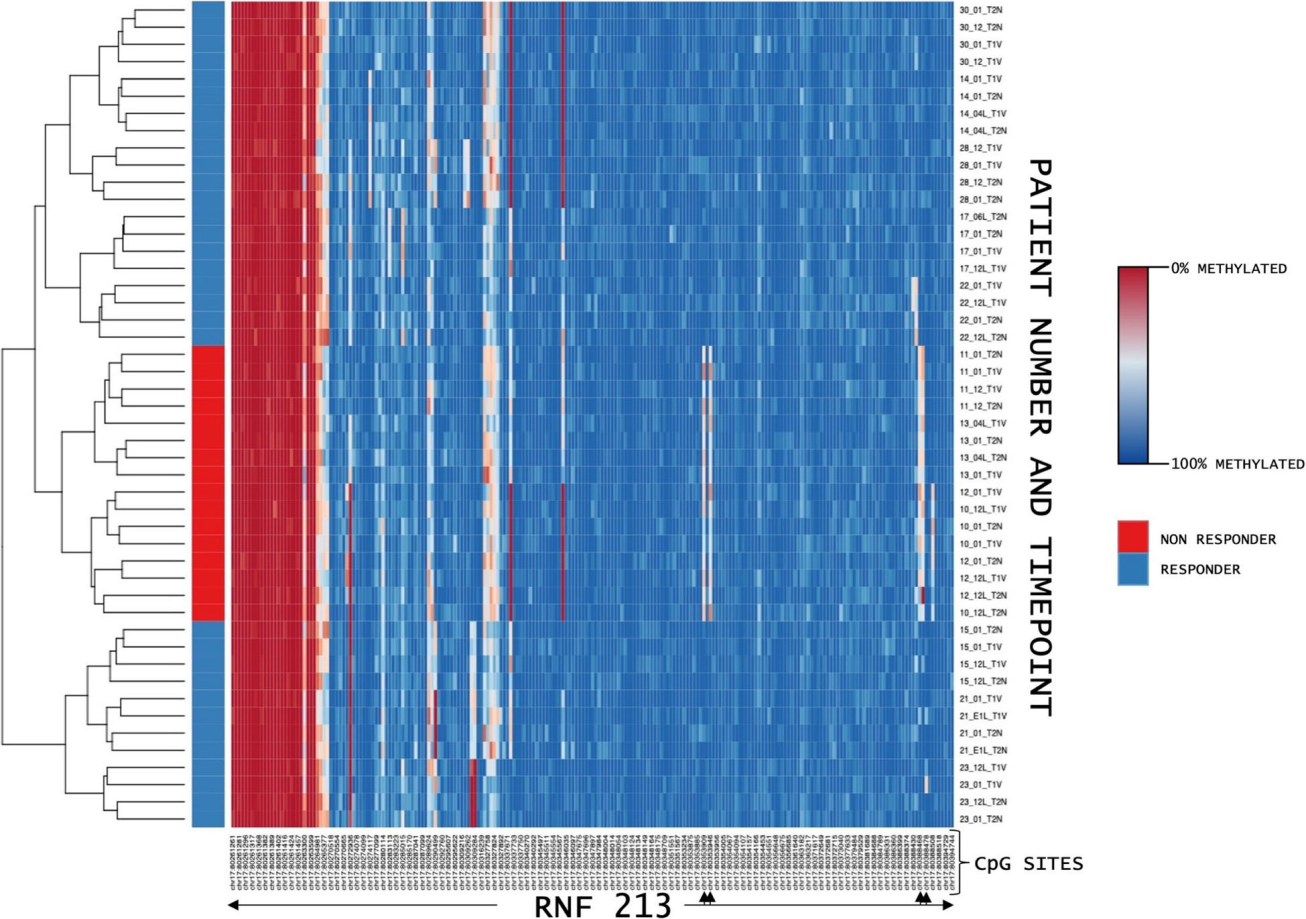
Comparison of DNA methylation values between ECT responder groups. ECT responders (blue, *n* = 8) and non-responders (red, *n* = 4) differed in their baseline DNA methylation at four single CpG sites (indicated by arrows) located within the ring finger protein 213 gene (*RNF213*). The horizontal rows represent distinct time points from each patient. Their DNA methylation values were clustered based on their similarity. T1: before the 1st ECT, T2: after the 1st ECT, T3: before the last ECT, T4: after the last ECT

**Fig. 2:**
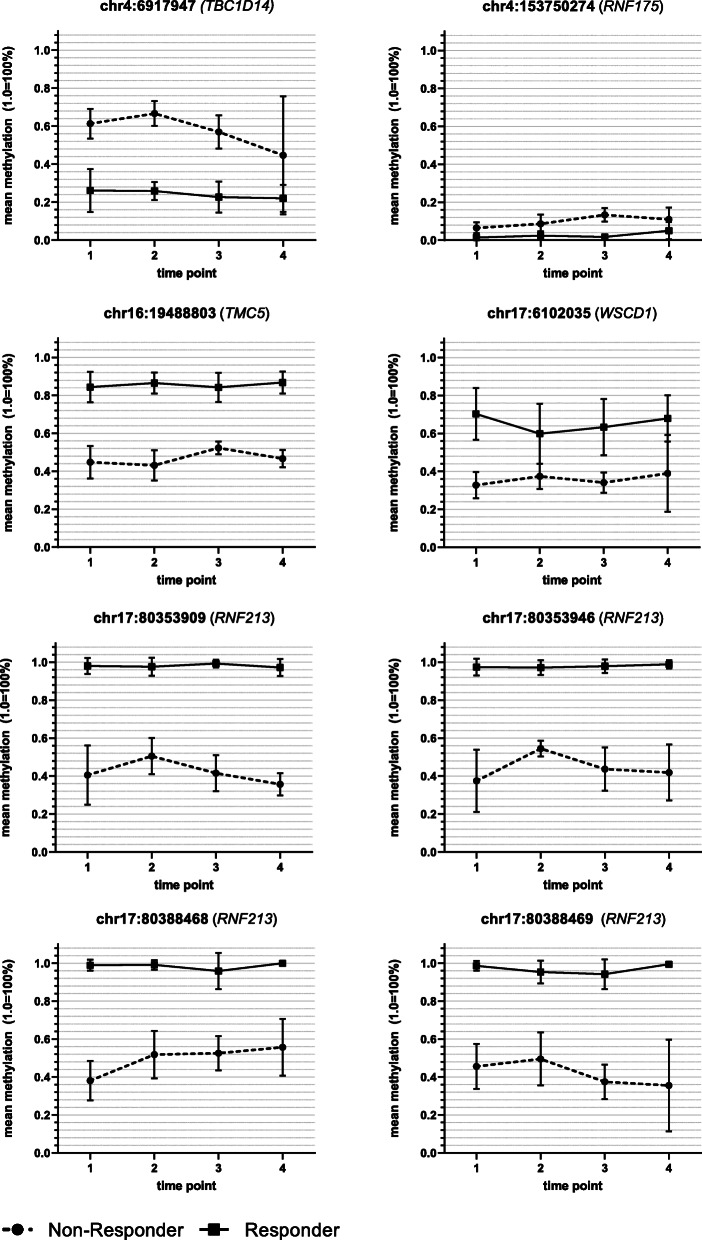
DNA methylation differences between ECT responder groups: protein-coding genes. DNA methylation of ECT responders (*n* = 8) and non-responders (*n* = 4) differed at eight CpG sites located within five different protein-coding genes: *TBC1D14* (=TBC1 domain family member 14), *RNF175* (=ring finger protein 175), *TMC5* (=transmembrane channel-like 5), *WSCD1* (=WSC domain containing 1), and *RNF213* (=ring finger protein 213). Time point 1: before the 1st ECT, 2: after the 1st ECT, 3: before the last ECT, 4: after the last ECT; error bars: ± SD

**Fig. 3:**
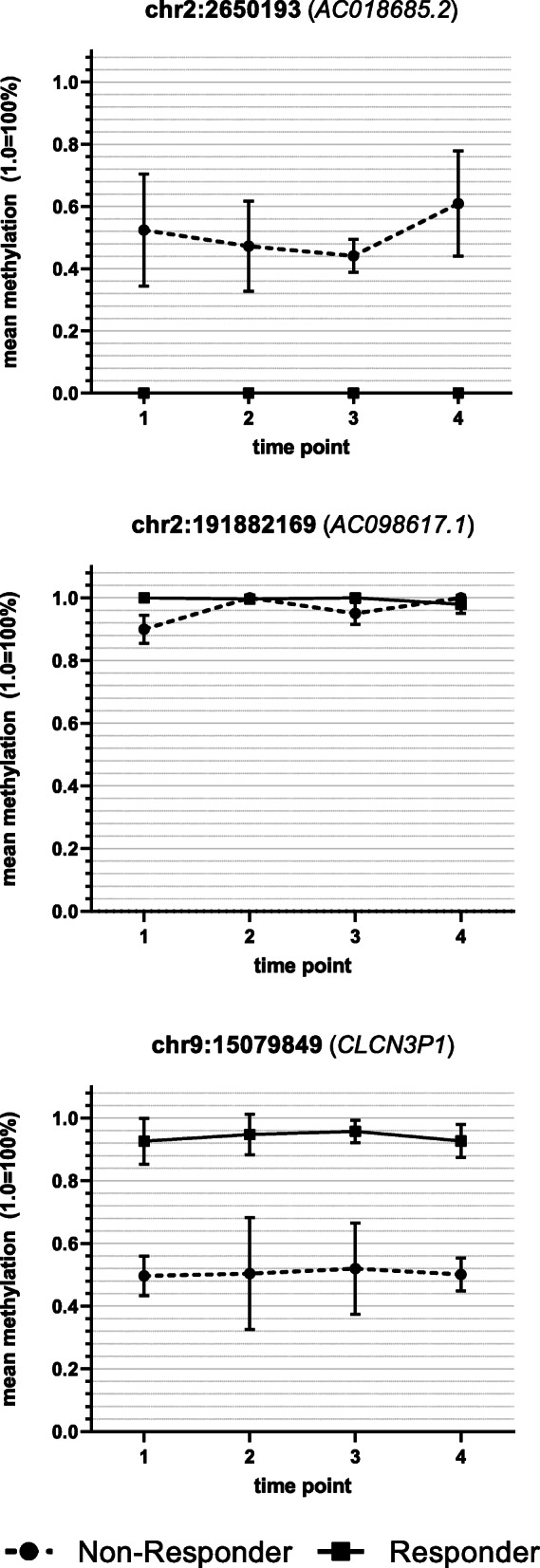
DNA methylation differences between ECT responder groups: long non-coding RNA transcripts. DNA methylation of three CpGs located within gene regions encoding for long non-coding RNA transcripts differed between ECT responders (*n* = 8) and non-responders (*n* = 4): *AC018685.2*, *AC098617.1*, and *CLCN3P1* (=chloride channel voltage-sensitive 3 pseudogene 1). Time point 1: before the 1st ECT, 2: after the 1st ECT, 3: before the last ECT, 4: after the last ECT; error bars: ± SD

**Fig. 4:**
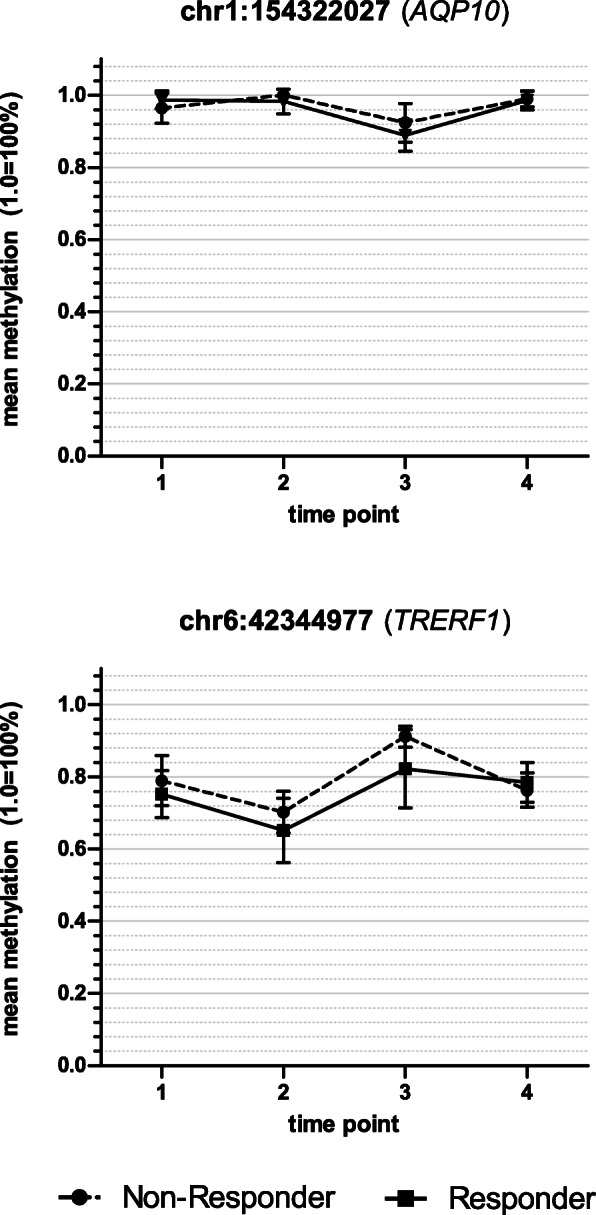
DNA methylation changes during the time course of ECT. DNA methylation of two CpG dinucleotides was found to change during the treatment course. These CpGs were located within the following genes: *AQP10* (=aquaporin 10) and *TRERF1* (=transcriptional regulating factor 1). Time point 1: before the 1st ECT, 2: after the 1st ECT, 3: before the last ECT, 4: after the last ECT; error bars: ± SD

Analysis of the global DNAm considering ECT response showed no significant effects for time (*F*(3, 30) = 2.37, *p* = 0.09), response (*F*(1, 10) = 0.05, *p* > 0.1) and the interaction between time and response (*F*(3, 30) = 0.14, *p* > 0.1). The analysis of variance for DNAm with respect to response/non-response for every probe (DMP) showed 13 significant probes located in ten different genes (seven protein-coding and three non-protein-coding (pseudo) genes (which encode for putative long non-coding RNA transcripts instead)) that met the previously established criteria of significance. A detailed presentation of the results regarding the 13 significant probes is shown in Table 2 and depicted in Figs. 1, 2, 3 and 4. Information regarding the genetic loci of our significant probes (and the genetic variants possibly affecting our CpGs of interest) was investigated using Ensembl [34], an internal JBrowse [35], GeneCards® [36], and the NHGRI-EBI GWAS Catalog [37].

**Table 2:** DNA methylation analysis—results

Gene	Gene location	CpG	Analysis of variance				SNP		MAF [1.0 = 100%]	
***AQP10***	chr1:154321116…154325325	chr1:154322027	Time	*F*(3, 30) = 17.22	*p* < 0.001	FD*R* = 0.04	rs1158005561	C > T	< 0.01	T
			Response	*F*(1, 10) = 0.47	*p* > 0.1	FD*R* = 1				
			Response × time	*F*(3, 30) = 1.61	*p* > 0.1	FD*R* = 0.98				
***RNF175***	chr4:153710125…153760235	chr4:153750274	Time	*F*(3, 40) = 3.11	*p* < 0.05	FD*R* = 0.92	rs562725996	G > A	0.0002	A
			Response	*F*(1, 40) = 46.08	*p* < 0.001	FD*R* < 0.001				
			Response × time	*F*(3, 40) = 1.92	*p* > 0.1	FD*R* = 0.98				
***RNF213***	chr17:80260866…80395312	chr17:80353909	Time	*F*(3, 40) = 2.32	*p* = 0.09	FD*R* = 0.93	rs34269699	G > A	0.04014	A
			Response	*F*(1, 40) = 729.15	*p* < 0.001	FD*R* < 0.001				
			Response × time	*F*(3, 40) = 2.21	*p* > 0.1	FD*R* = 0.98				
		chr17:80353946	Time	*F*(3, 40) = 2.28	*p* = 0.09	FD*R* = 0.93	rs11656211	C > T	0.03934	T
			Response	*F*(1, 40) = 538.15	*p* < 0.001	FD*R* < 0.001				
			Response x time	*F*(3, 40) = 2.71	*p* = 0.06	FD*R* = 0.98				
		chr17:80388468	Time	*F*(3, 40) = 2.71	*p* = 0.06	FD*R* = 0.93	rs1452340062	C > T	< 0.01	T
			Response	*F*(1, 40) = 415.46	*p* < 0.001	FD*R* < 0.001				
			Response × time	*F*(3, 40) = 2.87	*p* < 0.05	FD*R* = 0.98				
		chr17:80388469	Time	*F*(3, 40) = 1.22	*p* > 0.1	FD*R* = 0.97	rs6565682	G > A	0.1476	A
			Response	*F*(1, 40) = 340.88	*p* < 0.001	FD*R* < 0.001				
			Response × time	*F*(3, 40) = 1.61	*p* > 0.1	FD*R* = 0.98				
***TBC1D14***	chr4:6909242…6923771	chr4:6917947	Time	*F*(3, 40) = 2.56	*p* = 0.07	FD*R* = 0.93	rs7668673	C > T	0.2264	T
			Response	*F*(1, 40) = 88.62	*p* < 0.001	FD*R* < 0.001				
			Response × time	*F*(3, 40) = 1.17	*p* > 0.1	FD*R* = 0.99				
***TMC5***	chr16:19410539…19498140	chr16:19488803	Time	*F*(3, 30) = 0.16	*p* > 0.1	FD*R* = 0.97	rs16972066	C > T	0.07807	T
			Response	*F*(1, 10) = 9.99	*p* < 0.01	FD*R* = 0.02				
			Response × time	*F*(3, 30) = 3.31	*p* < 0.05	FD*R* = 0.98				
***TRERF1***	chr6:42225225…42452045	chr6:42344977	Time	*F*(3, 30) = 19.40	*p* < 0.001	FD*R* = 0.02	None		None	
			Response	*F*(1, 10) = 1.32	*p* > 0.1	FD*R* = 1				
			Response × time	*F*(3, 30) = 1.70	*p* > 0.1	FD*R* = 0.98				
***WSCD1***	chr17:6069106…6124427	chr17:6102035	Time	*F*(3, 40) = 0.32	*p* > 0.1	FD*R* = 0.98	rs59151763	G > A	0.1661	A
			Response	*F*(1, 40) = 51.20	*p* < 0.001	FD*R* = 0.001				
			Response × time	*F*(3, 40) = 0.56	*p* > 0.1	FD*R* = 0.99				
**Long non-coding RNA**										
***AC018685.2***	chr2:2638178…2696285	chr2:2650193	Time	*F*(3, 30) = 2.52	*p* = 0.08	FD*R* = 0.93	rs6719244	C/G/T	0.07688	C
			Response	*F*(1, 10) = 330.13	*p* < 0.001	FD*R* < 0.001				
			Response × time	*F*(3, 30) = 2.52	*p* = 0.08	FD*R* = 0.98				
***AC098617.1***	chr2:191846539…192044525	chr2:191882169	Time	*F*(3, 30) = 11.75	*p* < 0.001	FD*R* = 0.27	rs1455524094	G > A	< 0.01	A
			Response	*F*(1, 10) = 22.10	*p* < 0.001	FD*R* = 1				
			Response × time	*F*(3, 30) = 20.03	*p* < 0.001	FD*R* = 0.019				
***CLCN3P1***	chr9:14921013…15146401	chr9:15079849	Time	*F*(3, 30) = 0.26	*p* > 0.1	FD*R* = 0.99	rs10961870	G > A	0.2228	A
			Response	*F*(1, 10) = 229.65	*p* < 0.001	FD*R* < 0.001				
			Response × time	*F*(3, 30) = 0.03	*p* > 0.1	FD*R* = 1				

DNA methylation analysis of 1476812 single CpG sites (repeated ANOVA) revealed five novel protein-coding candidate genes (*RNF175*, *RNF213*, *TBC1D14*, *TMC5*, *WSCD1*) and three non-protein coding genes (*AC018685.2*, *AC098617.1*, *CLCN3P1*) to be implicated in ECT response. DNA methylation of two CpGs was found to change significantly during the treatment course (*AQP10*, *TRERF1*). All significant CpG sites (but one) do overlap with the loci of a listed single-nucleotide polymorphism (SNP)

As the duration of the current depressive episode differed between ECT responders (*n* = 8) and non-responders (*n* = 4) in the subgroup of patients analyzed (with ECT non-responders suffering from episodes more than twice as long), we conducted an additional correlation analysis by using a Spearman rank-order correlation test. None of our CpG sites reached statistical significance, though a tendency at the gene locus chr16:19488803 (*TMC5*) was present (rho = − 0.53, *S* = 437.53, *p* = 0.076); Figure S1).

## Discussion

DNAm analysis of 1476812 single CpG sites revealed five novel (protein-coding) candidate genes to be implicated in ECT response (*RNF175*, *RNF213*, *TBC1D14*, *TMC5*, and *WSCD1*). Further differences between ECT responder groups were found within gene regions encoding for long non-coding RNA transcripts (*AC018685.2*, *AC098617.1*, and *CLCN3P1*). In all cases (except one: *AC098617.1*), DNAm differed already at baseline and remained stable throughout the time course. Analyzing ECT’s effect irrespective of clinical outcome, DNAm of merely two CpG sites (located within *AQP10* and *TRERF1*) was found to change during the treatment. Intriguingly, all significant CpGs, but one (chr6:42344977, *TRERF1*), are known to overlap with a single-nucleotide polymorphism (SNP) directly located within these particular dinucleotides, generating or removing these CpGs and thus DNAm as a consequence. Due to the small group size of the current study, the results must be interpreted with caution, particularly due to the differences between ECT responder and non-responder groups. Nevertheless, the identified genes could be important candidates for therapeutic outcome prediction in future studies.

In this regard, the most striking difference in DNAm between ECT responder groups was present at four CpG sites located within the *RNF213* gene. Importantly, *RNF21*3’s DNAm has previously been reported to differ between MDD subjects and healthy controls, though in a much lower magnitude than in our cohort and without being comprehensibly corrected for multiple testing [38, 39]. The gene encodes for a homonymous 591-kDa protein (ring finger protein 213) that contains a RING finger domain mediating protein-protein interaction [40, 41]. Together with its postulated AAA+ ATPase and E3 ligase activity, RNF213 is enabled to unfold and link proteins to ubiquitin [41], a small 8.6-kDa protein whose linkage can lead to diverse outcomes depending on the particular amino acid it is bound to. Among ubiquitin’s various roles, its implication in the proteasome protein degradation system is one of the most pronounced [42–44]. By these means, RNF213 contributes to the clearance of two proteins involved in vascular remodeling via the Wnt signaling pathway [45]. Its striking role in vascular development is further supported by clinical studies revealing a particular *RNF213* mutant (p.R4859K, caused by a SNP of c.14576G>A) to be strongly associated with Moyamoya disease (MMD)—an occlusive cerebrovascular disorder that is marked by progressive stenosis, a concomitant formation of collateral vessels, and transient seizures [46, 47]. Intriguingly, ample evidence links angiogenesis to either MDD or its treatment. In this context, elevated vascular endothelial growth factor (*VEGF*) mRNA has been found in depressed subjects [48]. Further support for this notion stems from animal experiments, showing hippocampal angiogenesis to be boosted following electroconvulsive stimulation (ECS) [49]. Moreover, clinical neuroimaging studies report a particular SNP (rs699947, 2578C/A; located within the promoter region of *VEGF*) to be associated with hippocampal volume changes after ECT treatment [50].

As ECT has been demonstrated to have immunomodulatory properties [51–54] and to (partially) reverse the immunological irregularities found in MDD patients (or at least in a subgroup thereof) [55, 56], the immune system seems to serve as another link between the strong implication of *RNF213*’s DNAm and the clinical response to ECT. In this context, *RNF213* mRNA was found to be predominantly expressed in immunological tissue [46], and its expression to be enhanced upon pro-inflammatory stimulation [57]. In addition, RNF213 has been reported to affect the number of T regulatory cells [58], an immune cell population shown to be reduced in depressed subjects [59]. Finally, another connection between RNF213 and depression is formed by the let-7 family of miRNAs, i.e., short RNA sequences that were found to suppress the common variant of the *RNF213* gene. Within this context, particularly let-7c was shown to be either increased or diminished in MMD and MDD patients [60, 61].

According to our analysis, another ring finger protein (RNF175) has been linked to ECT response, though its function is less well characterized. Current studies suggest a SNP located within *RNF175* (rs981844) to be associated with the response to statins [62], i.e., a group of pharmaceuticals with suggested antidepressant properties [63]. However, despite this sparsity of literature, one thing is clear: RNF213 and RNF175 share their E3 ubiquitin-ligase activity [64], moving ubiquitin again into the spotlight of ECT responsiveness. Its outstanding role is further supported by several studies suggesting the DNAm of other ring finger proteins (as RNF138, RNF130 [65], and RNF2 [66]) to differ between the postmortem brain samples of depressed subjects and healthy controls, although these results do not reach statistical significance after type 1 error correction. However, besides its involvement in the proteasome degradation system, ubiquitin further leads to the removal of cellular structures by mediating selective autophagy [67].

Similarly, DNAm of the TBC1 domain family member 14 (*TBC1D14*) gene, a negative regulator of starvation-induced autophagy [68], was also found to differ in relation to clinical outcome, indicating ECT responsiveness to not be dependent on the selective type of autophagy alone. Intriguingly, a profound body of evidence illustrates autophagy to be crucially involved in depression [69]. Alcocer-Gómez et al., for instance, reported the expression of autophagy proteins to be upregulated in MDD patients [70]. Moreover, expression of autophagy proteins (i.e., Beclin-1 and light chain 3-II/I (LC3-II/I)) was shown to be elevated in the rat hippocampus following ECS treatment [71], leading Gassen and Rein to hypothesize that autophagic mechanisms (although already heightened at baseline) might still be insufficient in some disease cases [69]. In the context of ECT, boosted autophagic turnover—ensuring efficient recycling of amino and fatty acids and thus the production of urgently needed proteins [72, 73]—would fit well into the picture as an increase in glucose metabolism has been observed in several brain regions (like the hippocampus, for instance) following ECS treatment [74], indicating enhanced metabolic activity thus a higher demand for nutrients. However, the role of autophagy in MDD is still controversial, but the involvement of RNF213, RNF175, and TBC1D14 in cellular degradation hints at a role for these processes in ECT responsiveness, nevertheless.

The connection of the other genes found in our study (being differentially methylated in ECT responder groups) is less clear, yet no less interesting: An intergenic polymorphism (rs75213074) near the WSC domain containing 1 (*WSCD1*) gene has been previously associated with migraine [75], a neurological disorder sharing several biological abnormalities with depression [76]. Regarding transmembrane channel-like 5 (TMC5), a SNP (rs4780805) located − 17 kbp upstream of its gene was reported to correlate with sleeping duration [77], forming a link to MDD as sleeping patterns are often disturbed in depressed subjects [78].

With respect to ECT’s general effects (whether directly induced, a coincidence, or merely of secondary origin), DNAm of two CpG sites (located within aquaporin 10 (*AQP10*) and transcriptional regulating factor 1 (*TRERF1*)) were altered during the treatment course yet had no effect on clinical outcome. The former gene encodes for a water-permeable channel [79] that has not yet to our knowledge been linked to neuropsychiatric disorders. The same holds true for *TRERF1*, which regulates the expression of a mitochondrial enzyme (cytochrome p450 11A1–CYP11A1) that catalyzes the synthesis of pregnenolone, i.e., the substrate for all known steroids [80, 81]. Irregularities in glucocorticoids (a subtype of steroids) have been repeatedly reported in MDD patients [82], being modulated by ECT as robust rises in cortisol have been found following a single ECT session [83–85].

Despite this extensive body of evidence linking the latter mentioned genes to either MDD or ECT, several questions remain. In fact, the role of these particular genes within immune cells (the sample type, we obtained our measures from) is largely unclear: Regarding *TRERF1*, glucocorticoids are well known for their immunoregulatory properties, mediating diverse effects depending on their concentration [86, 87]. Ubiquitin and autophagy affect immunological processes at multiple points: Ubiquitin regulates signaling cascades involved in the activation of NF-кB, and thus, the subsequent production of pro-inflammatory cytokines [88], whereas autophagy has been reported to mediate anti-inflammatory functions by clearing accumulating proteins, apoptotic bodies, and pathogens [73]. Nevertheless, despite being generally involved in ubiquitin-linkage (*RNF213* and *RNF175*) and the negative regulation of autophagy (*TBC1D14*), it is largely uncertain whether these particular genes directly contribute to the latter mechanisms in immune cells as well, although indicated by some sources [89, 90].

A clear interpretation of the data is further hindered by the insufficient knowledge of the genetic regulation of the proposed candidate genes. In fact, the consequence of the DNAm differences or changes in the expression of the proposed genes is unknown and can only be assumed on the basis of the current literature. In this context, most of the significant CpG sites are located within introns, i.e., regions which were found to be generally low in CpGs. If prevalent, DNAm of these CpGs was suggested to modulate alternative splicing [91, 92], to suppress transposable elements [93], or to regulate the usage of alternative promoters [91, 94]. Hence, DNAm of these specific loci does not ultimately indicate the inhibition of gene expression (as it has been suggested for promoter regions [91]) but might exert various roles. Some significant CpG sites were also found outside gene bodies, i.e., within promoter flanking regions, but the effect on gene expression is also rather unpredictable at these loci.

We furthermore cannot estimate the contribution of the SNPs located at our candidate loci that are either generating or removing the CpGs and thus DNAm as a consequence. Importantly, recent studies propose DNAm to interact with its underlying genotype (even if the respective CpG sites and SNPs are far apart) [95, 96] and the interaction of genetic and environmental factors to be particularly relevant for disease risk [3, 33, 95]. In the case of our SNPs, their minor allele frequency (MAF) values (see Table 2), indicating the second most common variant at a defined locus, are generally relatively small and the removal of CpGs at these particular sites therefore rather unlikely, but not entirely out of question.

The interpretation of the biological significance of our results is further restricted by the sparsity of studies using the TruSeq Methyl Capture EPIC Library Kit for DNAm analyses. Instead, most researchers use either the Illumina Infinium Human Methylation 450 K or the Infinium MethylationEPIC 850 K BeadChip for their experiments. Because both microarrays do not cover our proposed candidate CpG sites, a comparison to the DNAm of other MDD cohorts or healthy subjects is, unfortunately, unfeasible at these particular loci. Consequently, we are also unable to assess the influence of the patients’ clinical characteristics on DNAm other than in our cohort. In this regard, we found a tendency for the DNAm of chr16:19488803 (*TMC5*) to be influenced by the patient’s respective current episode duration. Since smoking behavior has also differed greatly between ECT responders (five smokers) and non-responders (one smoker), evaluating its influence on DNAm levels would have been of interest but was not feasible due to the small sample size. Studying the literature regarding its general influence on DNAm (not looking at our particular loci of interest only), we found *TRERF1’s* DNAm (chr6:42219847) to be affected by tobacco intake [97]. Altogether, we thus cannot entirely rule out a possible impact of the patients’ clinical characteristics on our DNAm analysis.

Further, as MDD is primarily thought to be caused by a malfunctioning of neuronal processes, analyzing the correlation between the DNAm in the brain and the periphery would have been of great value. Unfortunately, the web tools frequently used for this investigation (as BECon [98] or BloodBrain [99]) are based on data obtained from the Illumina 450 K array and are thus not applicable to our results. However, as all of our candidate CpGs (except one) do overlap with a known SNP, the observed DNAm difference between ECT responders and non-responders might be a result of their genotype rather than being a pure epigenetic difference. The extent of their DNAm difference and the stability of its pattern during the treatment course supports this idea. In this case, a high correlation between blood and brain DNAm would be rather likely.

Despite these limitations—with the small group size being the most restricting one—our study might be of great value for future approaches, as data generated by the TruSeq Methyl Capture EPIC Kit is still a rarity, especially in the context of MDD.

## Conclusions

DNAm of 13 single CpG sites (located within ten genes encoding for either a protein or a long-coding RNA transcript) was found to differ between ECT responder groups or to alter within the treatment course of ECT. The data of the current work thus provides a deeper insight into ECT-associated effects and suggests novel candidate genes for ECT response prediction. Due to a small sample size, the findings must be regarded as preliminary; a replication in larger cohorts is required.

## Methods

### Study design

Our cohort of depressed ECT patients (*n* = 17) was acquired at the Department of Psychiatry, Social Psychiatry and Psychotherapy at the Hannover Medical School (Germany). The study complies with the ethical principles of the Declaration of Helsinki (1964, including its later amendments) and was approved by the Ethics Committee of the Hannover Medical School (NEKTOR-Registry: 2842-2015). Written informed consent was signed by all participants prior to study inclusion. As this is a naturalistic long-term observational study of one patient cohort, participants were grouped according to their clinical course (to either ECT responder or non-responder) after treatment completion.

### P**atients**

MDD diagnosis was established using the International Statistical Classification of Diseases and Related Health Problems 10th Revision (ICD-10) and depression severity assessed via two psychometric questionnaires, namely the Beck Depression Inventory (BDI-II) and the Montgomery-Åsberg Depression Rating Scale (MADRS). However, only the latter test served for the assignment of patients to clinical outcome groups. In this context, a decrease of ≥ 50% in MADRS scores was interpreted as a treatment response. The Mini-Mental State Examination (MMSE) was conducted at the same time points (i.e., at baseline and after the first and last ECT). Patients who had an autoimmune, infectious, or schizophrenic disorder were excluded from our study. Heightened levels of CRP, a prominent leukocytosis, or medication with immunomodulatory drugs were additional exclusion criteria.

### Application of ECT and sample collection

During the actual treatment course, ECT was applied three times weekly for up to 4 weeks, followed by maintenance ECTs applied only once a week. Right unilateral electrical stimulation was performed using an ultra-brief impulse device (Thymatron® System IV, Somatics, LLC). The seizure threshold was assessed at the first ECT session (based on an age-dependent method), and the stimulus intensity adjusted according to the recorded motoric and electroencephalographic (EEG) seizure duration. If the patient did not show any improvement of symptoms after two following weeks of treatment, bilateral electrode placement was considered. During ECT, patients were anesthetized with methohexital and remifentanil while muscle relaxation was achieved with succinylcholine. Fasting blood samples were taken at four different time points, namely directly before (i.e., 8 a.m.–10 a.m.) and 15 min after the first and the last ECT session, respectively. Samples were stored at 4 °C until further processing (3 hours maximum).

### Sample processing

#### PBMCs—isolation and thawing

PBMCs were isolated by gradient centrifugation as described elsewhere [100]. Based on the recommendations of Mallone et al. [101], changes have been applied to the latter procedure. After isolation, PBMCs were kept at − 196 °C until thawing. Thawing was performed according to a protocol published by the Helmholtz Institute in Munich [102], though (as in the previous case) adaptions were made to meet our requirements. A detailed description of all steps performed to isolate, freeze, and thaw PBMCs is provided in the supplements.

#### HeLa—thawing and splitting

HeLa cells (immortal cervical cancer cells) were required as a quality control for sequencing. For this purpose, early passage cells were thawed and split according to a protocol published elsewhere [103].

#### DNA isolation

Genomic DNA (gDNA) of PBMCs and HeLa cells was isolated using the AllPrep DNA/RNA 96 kit (#80311; QIAGEN N.V.). Minor changes have been made to the recommended procedure; a detailed description is to be found in the supplements. After isolation, gDNA of patients was kept at − 80 °C and gDNA of HeLa cells at − 4 °C (being supplemented with 0.5 mM UltraPure^TM^ EDTA (#11568896; Invitrogen AG) in the latter case; storage duration: maximum 3 weeks).

#### L**ibrary generation, quality control, and quantification**

Five hundred nanograms of total gDNA per sample was pipetted into microTUBE AFA Fiber Pre-Slit Snap-Cap 6x16mm tubes (#520045; Covaris, Inc.) and subsequently sheared using a Covaris S220 Ultrasonicator. The sheared gDNA was utilized as input for preparing targeted methylseq libraries with the TruSeq-Methyl Capture EPIC Library Kit (#FC-151-1003; Illumina, Inc.), allowing a preparation of up to 48 libraries at four-plex within less than 2 days. All steps were performed as recommended in the Illumina user document 1000000001643 v01 May 2017, though one additional purification step was introduced at the end of the standard procedure, using 1× Agencourt® AMPure® XP Beads (#A63881; Beckman Coulter, Inc.). Four-plex DNA samples were barcoded by a single indexing (6 bp) approach using 12 different DNA indexes. All generated DNA libraries were amplified with 11–13 cycles of PCR using a KAPA HiFi HotStart Uracil+ Ready Mix (2X) enzyme (#KK2801; Kapa Biosystems), which was not included in the kit. Fragment length distribution of individual libraries was monitored using the Bioanalyzer High Sensitivity DNA Assay (#5067-4626; Agilent Technologies). Quantification of libraries was performed by the use of the Qubit® dsDNA HS Assay Kit (#Q32854; ThermoFisher Scientific). Importantly, gDNA of HeLa cells served as a quality control and was thus added to each run of library preparation and subsequent sequencing (1–2 aliquots per run).

#### Library denaturation and sequencing run

Equimolar amounts of twelve individually barcoded libraries were pooled. Each analyzed library relevant to this project constitutes 8.7% of an overall flow cell capacity. The library pool was denatured with sodium chloride (NaOH, #72082-100 ml; SIGMA-ALDRICH Co.) and was finally diluted to 1.8 pM according to the Denature and Dilute Libraries Guide (Document #15048776 v02; Illumina, Inc.). 1.3 ml of the denatured pool was loaded on an Illumina NextSeq 550 Sequencer using a High Output Flow Cell for paired-end reads (Document #20024907; Illumina, Inc.). Paired-end sequencing was performed with 76 cycles, a 6-base barcode index, and 25% calibration control v3 PhiX library (#FC-110-3001; Illumina, Inc.). This level of PhiX was required as the samples were relatively GC rich. In total, 8 NextSeq runs were performed.

### Data processing

#### Sequence data analysis

Illumina 75 bp paired-end datasets were demultiplexed using bcl2fastq version 2.17.1.14 (Illumina, Inc.). Fastq files were then subjected to quality control with FASTQC and MultiQC [104] and analyzed using the nf-core methylseq pipeline with the Bismark software (version 1.5dev) [105, 106]. The genome reference hg38 from Ensembl was used without decoy sequences. The pipeline was modified to only make methylation calls for sites covered by at least 5 reads, instead of the default 1 read. Coverage and methylation calls were converted into bigwig format and visualized in the JBrowse web application [35]. All data were analyzed on the MHH HPC-seq SLURM research cluster. Where several runs were necessary to achieve sufficient coverage, FASTQ files were combined before analysis.

#### Quality control

In order to control for technical variability (which was necessary as our experiment included several runs of library preparation and sequencing), a detailed quality control of the measurement was carried out. In this context, isolated gDNA from HeLa cells was added to each sequencing run. The resulting average HeLa-cell-probe correlation (whose calculation included all CpG sites of all measurements with a previously defined minimum coverage of least 5 reads) was *M*_*r*_ = 0.97 and based on 14 measured samples and 2016707 observed CpG probes each, indicating a sufficiently good accuracy of our measurements. Furthermore, the measured samples were examined with density plots and dendrograms. One patient sample showed a conspicuous value distribution pattern and was different from all other samples in the cluster analysis. Therefore, a faulty measurement was assumed, leading to the subsequent exclusion of this sample from further analyses. In order to avoid gender bias, chromosome X and Y were additionally excluded. After all quality control measures, 1476812 CpG probes (per sample) were examined for final statistics.

### Statistical analyses

Demographics of patients were normally distributed. *T* tests and Fisher’s exact tests were used for the analysis of demographic, psychometric, and other clinical baseline differences between ECT responders and non-responders. Regarding the methylation analysis of our cohort, we first checked for ECT-associated changes considering the overall DNAm levels (=mean of all measured CpG sites), being investigated with repeated measures ANOVA modeling approach implemented in the *lmer* package [107] and separated for “response” as an outcome definition. Second, we performed a detailed analysis for the differences in methylation with respect to response/non-response for every probe (DMP) with a series of repeated measures ANOVA. As a correction for multiple testing, a false discovery rate (FDR) of < 5% in combination with a minimum variance of 0.1 was defined as significant. Only samples with complete data at all four time points (*n* = 12) were included in the analyses. Statistical analyses of the patients’ clinical baseline characteristics were performed using IBM SPSS Statistics 25.0 for Windows (IBM Corp.), the methylation analyses were conducted within the R (3.6.1) environment on Windows 10.0.18362.

## Supplementary information

**Additional file 1.**

## Data Availability

The datasets used and/or analyzed during the current study are available from the corresponding author on reasonable request because they include genetic information of patients.

## Abbreviations

AQP10: Aquaporin 10
BDI-II: Beck Depression Inventory
BDNF: Brain-derived neurotrophic factor
BMI: Body mass index
COMT: Catechol-O-methyltransferase
CpG: Cytosine-phosphate-guanine dinucleotide
CRP: C-reactive protein
CYP11A1: Cytochrome p450 11A1
DMP: Differences in methylation for every probe
DNAm: DNA methylation
DRD2: Dopamine receptor D2
ECS: Electroconvulsive stimulation
ECT: Electroconvulsive therapy
EEG: Electroencephalography
FDR: False discovery rate
gDNA: Genomic DNA
HPA: Hypothalamic-pituitary-adrenal
ICD-10: International Statistical Classification of Diseases and Related Health Problems 10th Revision
LC3-II/I: Light chain 3-II/I
MADRS: Montgomery-Åsberg Depression Rating Scale
MAF: Minor allele frequency
MDD: Major depressive disorder
MMD: Moyamoya disease
MMSE: Mini-Mental State Examination
NaOH: Sodium chloride
PBMCs: Peripheral blood mononuclear cells
RNF175/213: Ring finger protein 175/213
SD: Standard deviation
SNP: Single-nucleotide polymorphism
TBC1D14: TBC1 domain family member 14
TMC5: Transmembrane channel-like 5
TRERF1: Transcriptional regulating factor 1
VEGF: Vascular endothelial growth factor
WSCD1: WSC domain containing 1
