# Supplementary material for: Novel candidate genes for ECT response prediction—a pilot study analyzing the DNA methylome of depressed patients receiving electroconvulsive therapy
Source: Clin Epigenetics. 2020 Jul 29;12:114. doi: 10.1186/s13148-020-00891-9 (PMC7388224; doi:10.1186/s13148-020-00891-9)
Supplement: Supplementary file 1 — Additional file 1. [file 13148_2020_891_MOESM1_ESM.pdf]

SUPPLEMENTARY MATERIAL

METHODS

Sample Processing

PBMC Isolation

PBMCs were isolated by density centrifugation as described by Sieve et al. [100], though (based on the recommendations of Mallone et al. [101]) adaptions were made to meet our requirements. For the present work, PBMCs were isolated as follows: 17 ml of EDTA-blood was centrifuged (2000xg, 10 min, room temperature (RT)) and 4 ml of the resulting supernatant (=plasma) stored for other experiments. The removed volume was substituted with 4 ml of Dulbecco's Phosphate Buffered Saline (PBS, #L1825; Biochrom GmbH) and the monovettes inverted before transferring the blood into 50 ml tubes (#62.547.004; SARSTEDT AG & Co. KG). Afterward, monovettes were washed with PBS twice and further PBS added until a 1:2 dilution of the original blood volume was reached. Diluted blood was layered onto 12 ml of Biocoll® Separating Solution (#L6113; Biochrom GmbH) and subsequently centrifuged (2000xg, 20 min, RT) with the deceleration speed set to zero. The supernatant was removed (up to 5 ml above the buffy coat) and the cells transferred into a new 50 ml conical tube. PBMCs were washed with cold (4°C) PBS (supplemented with 1% bovine serum albumin (BSA, #A1470; SARSTEDT AG & Co. KG)) for three times (1200xg, 10 min, 4°C) before counting. For quantification, nonviable cells were marked with 0.2% trypan blue (#T6146; SIGMA-ALDRICH Co.), pipetted into a Neubauer chamber (#0640010; Paul Marienfeld GmbH & Co. KG) and visualized using an inverted CKX41 microscope (Olympus). After a final centrifugation step (1200xg, 10 min, 4°C), freshly prepared freezing medium (containing 10% dimethyl sulfoxide (DMSO, #41640-M; SIGMA-ALDRICH Co.) and 10% human serum (of the patient and time point, respectively) in PBS) served for the resuspension of the resulting cell pellet. Resuspended cells were then aliquoted (10-20x10^6 cells/ml; CryoPure Tubes (#72.380; SARSTEDT AG & Co. KG)), put into pre-cooled (4°C) Mr. Frosty™ freezing containers (#5100-0001; Thermo Fisher Scientific; filled with isopropanol) and stored at -80°C for up to 72 h. To assure optimal preservation of the cells, PBMCs were kept in a liquid nitrogen tank (MVE Cryosystem 750, Jutta Ohst german-cryo® GmbH) until further processing.

PBMC Thawing

For the current work, PBMCs were thawed by keeping the cryotubes (that contained the cells) in a 37°C-warm water bath until only a small bit of ice remained. 1 ml of heated RPMI 1640 medium (#FG1215; Biochrom GmbH; 37°C, supplemented with 2% BSA) was subsequently added to each sample. Thawed PBMCs were then transferred into 50 ml tubes that contained 5 ml of the 2%-BSA-RPMI-medium (37°C). Afterward, 10 ml of the medium was additionally added. Cells were subsequently centrifuged (900xg, 5 min, 4°C) and fluorescence-activated cell sorting (FACS) buffer (=PBS supplemented with 1% BSA and

1mM UltraPure^{TM} EDTA (#11568896; Invitrogen AG)) used for washing the cells for three times. PBMCs were then stored on ice for 30 min and manually quantified (0.2% trypan blue). The suspension was subsequently divided into 5 ml polypropylene tubes (#55.526.006; SARSTEDT AG & Co. KG), centrifuged (900xg, 5 min, 4°C) and the supernatant discarded. The cells were resuspended in 500 µl RNAprotect Cell Reagent (#76526; QIAGEN N.V.) and kept at 4°C until further processing (up to one week).

Genomic DNA Isolation from PBMCs and HeLa cells

gDNA of PBMCs and HeLa cells was isolated using the AllPrep DNA/RNA 96 kit (#80311; QIAGEN N.V.). Minor changes have been applied to the manufacturer's instructions. In short, cells were centrifuged at 5000xg, 4°C for 5 min before isolation. The supernatant was discarded and 300 µl of RLT buffer (containing 1% β-Mercaptoethanol (#A1108; AppliChem GmbH)) added to each sample. Cell lysis was obtained by pulse vortexing (2x30 sec, 4°C) and subsequent storage on ice for at least 15 min. The lysates were pipetted into the wells of an AllPrep96 DNA plate, centrifuged (5600xg, 4 min, RT), and washed thrice (1x AW1 buffer, 2x AW2 buffer). For gDNA elution, each sample was incubated with 50 µl of heated (70°C) EB buffer for 5 min and subsequently centrifuged (4 min at 5600xg, RT). The elution was repeated, and the combined eluate stored at -80°C (PBMCs) or 4°C (HeLa cells) until further processing.

RESULTS

Patients' Clinical Baseline Characteristics (n=12)

Final DNAm analyses were performed using data from 12 patients only. After treatment completion, 8 responded to ECT. Three patients had minimal heightened levels for leukocytes (11.2-12.4x10^3/µl), but no signs of infection (i.e., elevated CRP). During ECT, patients were anesthetized with methohexital (mean=128.3(±57.8) mg, minimum=90 mg, maximum=250 mg) and remifentanil (89.6(±58.2) mg, 30 mg, 200 mg) and received succinylcholine for muscle relaxation (121.7(±52.0) mg, 60 mg, 200 mg). Responders and non-responders differed only in their numbers of total leukocytes (t-test, p=0.048, T=-2.249) and the duration of their current episode (t-test, p=0.026, T=2.948).

**Patients for final analysis**		Whole cohort (n=12)	Responder (n=8)	Non-responder (n=4)
**Demographics**				
**Age in years**, mean (±SD; range)		53.6 (±14.7; 24–70)	58.5 (±10.3; 43–70)	43.8 (±18.8; 24–67)
**Gender**, n (%)	female	7 (58.3%)	6 (75.0%)	1 (25.0%)
	male	5 (41.7%)	2 (25.0%)	3 (75.0%)
**Body mass index**, mean (±SD; range)		27.1 (±4.9; 20–39)	28.4 (±4.8; 23–39)	24.5 (±4.8; 20–30)
**Smokers**, n (%)	yes	6 (54.5%)	5 (71.4%)	1 (25.0%)
**Psychometric characteristics**				
**Age at diagnosis in years**, mean (±SD; range)		34.5 (±12.2; 18–53)	36.3 (±12.2; 21–53)	31.8 (±13.5; 18–49)
**Current episode in weeks**, mean (±SD; range)		33.6 (±21.2; 3–68)	24.8 (±15.2; 3–48)*****	60.0 (±11.3; 52–68)*****
**BDI**, mean (±SD; range)		37.4 (±11.9; 16–56)	37.1 (±12.9; 16–56)	38.0 (±11.5; 24–52)
**MADRS**, mean (±SD; range)		29.4 (±10.0; 12–43)	30.5 (±13.0; 12–43)	27.8 (±3.5; 24–32)
**MMSE**, mean (±SD; range)		28.2 (±2.8; 21–30)	27.7 (±3.4; 21–30)	29.0 (±1.4; 27–30)
**Psychotic symptoms**, n (%)	yes	4 (33.3%)	3 (37.5%)	1 (25.0%)
**Suicidality**, n (%)	yes	2 (16.7%)	0 (0.0%)	2 (50.0%)
**Medication**				
**Antidepressant drugs**, n (%)	yes	12 (100.0%)	8 (100.0%)	4 (100.0%)
**Benzodiazepines**, n (%)	yes	7 (58.3%)	6 (75.0%)	1 (25.0%)
**Antipsychotic drugs**, n (%)	yes	8 (66.7%)	6 (75.0%)	2 (50.0%)
**Lithium**, n (%)	yes	2 (16.7%)	1 (12.5%)	1 (25.0%)
**Clinical parameters**				
**Leukocytes in x10^3/µl**, mean (±SD; range)		7.5 (±2.3; 4.3–12.4)	8.4 (±2.2; 6.5–12.4)*	5.7 (±1.2; 4.3–7.1)*

Table S1: ***Patients' clinical baseline characteristics (n=12).*** Clinical baseline characteristics of treatment-resistant MDD patients undergoing a course of ECT (whole cohort vs. responders/non-responders), presented as mean (±standard deviation (SD); range (=minimum – maximum)) or quantity (absolute and percentual, n (%)); *****p<0.01

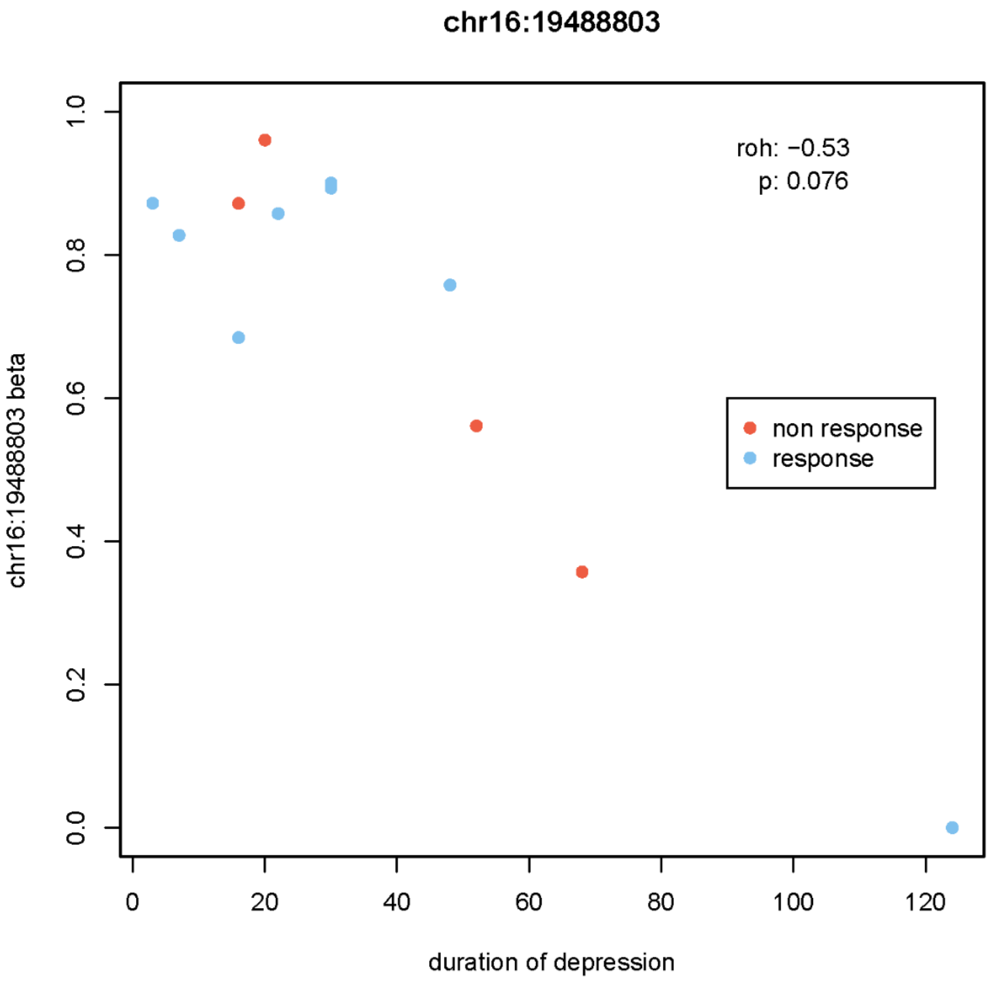

Figure S1: ***Influence of the Current Episode Duration on DNA Methylation.*** As the duration of the current depressive episode differed between ECT responders (n=8) and non-responders (n=4) in the subgroup of patients analyzed (with ECT non-responders suffering from episodes more than twice as long), we conducted an additional correlation analysis by using a Spearman rank-order correlation test. None of our CpG sites reached statistical significance, though a tendency at the gene locus chr16:19488803 (*TMC5*) was present (rho=-0.53, S=437.53, p=0.076).
